# Enhanced Mutant Screening in One-step PCR-based Multiple Site-directed Plasmid Mutagenesis by Introduction of Silent Restriction Sites for Structural and Functional Study of Proteins

**DOI:** 10.1186/s12575-017-0062-5

**Published:** 2017-09-26

**Authors:** Ting-Yu Kuo, Chung-Che Tsai, Hua-Wen Fu

**Affiliations:** 1 Institute of Molecular and Cellular Biology, National Tsing Hua University, Hsinchu, 30013 Taiwan, Republic of China; 2 Department of Life Science, National Tsing Hua University, Hsinchu, 30013 Taiwan, Republic of China

**Keywords:** Site-directed mutagenesis, Polymerase chain reaction, PCR, Silent restriction mutation, *Helicobacter pylori* neutrophil-activating protein, HP-NAP

## Abstract

Site-directed mutagenesis (SDM) has been widely used for studying the structure and function of proteins. A one-step polymerase chain reaction (PCR)-based multiple site-directed plasmid mutagenesis method with extended non-overlapping sequence at the 3′ end of the primer increases the PCR amplification efficiency and the capacity of multi-site mutagenesis. Here, we introduced silent restriction sites in the primers used in this PCR-based SDM method by utilizing SDM-Assist software to generate mutants of *Helicobacter pylori* neutrophil-activating protein (HP-NAP), whose gene has low GC content. The HP-NAP mutants were efficiently generated by this modified mutagenesis method and quickly identified by a simple restriction digest due to the presence of the silent restriction site. This modified PCR-based SDM method with the introduction of a silent restriction site on the primer is efficient for generation and identification of mutations in the gene of interest.

Site-directed mutagenesis (SDM) is a powerful technique to examine the effects of changing specific amino acid residues of a protein. The changes in biological and catalytic activity or stability of the mutated protein compared to its wild-type protein allow us to identify the residue that plays a critical role in the function of a protein. This technique is also essential in protein engineering [1]. Many SDM methods being developed are based on the polymerase chain reaction (PCR) [2–6]. The QuikChange SDM method is probably the most popular one among them [5]. With this approach, the mutation is introduced into a double-stranded plasmid with one pair of complementary primers containing the mutation of interest by a single-round PCR. To avoid the primer-dimer formation, the melting temperature (Tm) and GC content of the primer need to be at least 78 °C and 40%, respectively, (QuikChange™ Site-Directed Mutagenesis Kit, Instruction Manual). This requirement complicates the primer design. For genes with low GC contents, it is almost impossible to design the complementary primer with a high Tm and a high GC content. A method with new primer design by the addition of short non-overlapping ends to the 3′ end of the primer pairs was developed to overcome this problem [7]. This design with partial overlapping primers reduces the primer-dimer formation and increases primer annealing to the template during the PCR [7]. To eliminate the primer dimerization and permit the newly synthesized PCR product to be used as the template for the next round of amplification, Liu and Naismith developed a modified method by further extending the non-overlapping sequence at the 3′ end of the primers to make the Tm of the non-overlapping sequences (Tm_no_) 5 to 10 °C higher than the Tm of the primer-primer complementary sequences (Tm_pp_) [8]. In this method, Tm_no_ – 5 °C is used as the annealing temperature during the amplification cycle, and an additional annealing step at Tm_pp_ – 5 °C is added before the final extension step to increase the synthesis of the full-length plasmid [8]. This method is not limited to introducing single site mutations, but can also be used in introducing deletion, insertion, and multiple-site mutations without extra steps.

During the course of our study, we encountered a problem in designing the primers to generate *Helicobacter pylori* neutrophil activating protein (HP-NAP) mutants using the QuikChange SDM protocol because the GC content of *napA* gene is lower than 40%. The PCR-based multiple site-directed plasmid mutagenesis method developed by Liu and Naismith was chosen for generating HP-NAP mutants. Although this method is quite simple and efficient, the unmutated parental clone could still be recovered [8]. Here, we applied SDM-Assist software [9] to introduce additional silent restriction mutations in the primers using in the SDM method developed by Liu and Naismith to generate mutations in HP-NAP for its functional study. The clones with desired mutations can be first identified by a restriction enzyme digest prior to sequence verification. The principle of this modified PCR-based SDM method with introduction of silent restriction sites in the primer is depicted in Fig. 1. The desired mutation and the silent restriction site mutation can be introduced at either the primer-primer complementary sequences or the non-overlapping sequences (Fig. 1a). The plasmid pET42a-NAP [10], which was used as the template to generate HP-NAP mutants, was first analyzed by a web server NEBcutter (http://nc2.neb.com/NEBcutter2/) to identify the restriction enzyme cleavage sites present on it and the cut frequency of the identified enzymes. The DNA sequence of the *napA* gene [GenBank: AE000543.1, Gene: HP0243] from *H. pylori* strain 26,695 was subjected to SDM-Assist software to design the primer with the desired mutations and silent restriction site mutations. The silent restriction sites were selected from those ones present in the final PCR products no more than two times. For generation of the five HP-NAP mutants with a single amino acid change, two to three mutated nucleotides were introduced into the primers. Except for the one mutated nucleotide of HP-NAP_E103D_ mutant being introduced at the non-overlapping sequences, all the other mutated nucleotides were introduced at the primer-primer complementary sequences. For generation of HP-NAP_E97GY101H_ mutant with a double amino acid change, four mutated nucleotides were introduced into the primers. Three of them were introduced at the primer-primer complementary sequences and one of them was introduced at the non-overlapping sequences. The details of the mutagenesis primers are shown in Table 1. All the primers were synthesized by Genomics BioSci & Tech (Taipei, Taiwan).

**Fig. 1:**
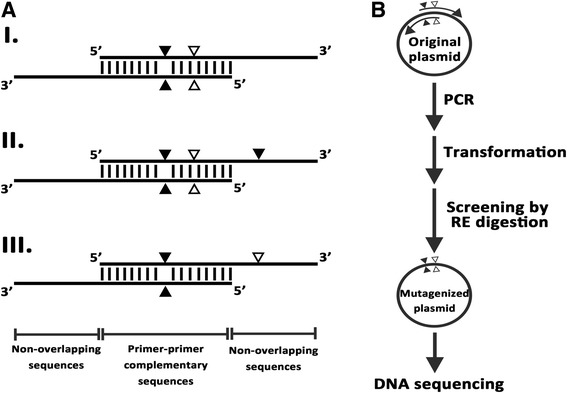
Schematic diagram of our modified PCR-based SDM method with introduction of restriction site on the primer. **a** Design of the mutagenic primers. The desired mutation and silent restriction site mutation can be introduced at the primer-primer complementary sequences or the non-overlapping sequences of the primers as shown in I, II and III. *Black* and *white* triangles indicate the locations of the desired mutation and silent restriction site mutation in the primer. **b** Flow chart of the procedures for mutagenesis and screening. The original plasmid is subjected to PCR using the primer pairs shown in **a**. The PCR products are transformed into *E. coli* and the plasmids isolated from the transformed colonies are subjected to silent restriction enzyme (RE) digestion for mutant screening. The mutations present in the plasmid identified by RE digestion are further confirmed by DNA sequencing

**Table 1:** Primers Used for Mutagenesis

HP-NAP mutants	Primer sequence (5′-3′)^a, b, c^	Tm_pp_ (°C)^d, e^	Tm_no_ (°C)^d, f, g^	Inserted silent restriction sites	Size of the digested products (bp)^h^
HP-NAP_D98A_	**F: AAATT** **CTcGAG** **GctTACAAA** TATCTAGAAAAAGAATTTAAAGAGC	54	62	XhoI	5261, 307
	**R: TTTGTAagC** **CTCgAG** **AATTT** CTTTAAAGATGTCTTTAGAGTGG	54	62		
HP-NAP_Y99A_	**F: ATT** **CTcGAG** **GACgcCAAA** TATCTAGAAAAAGAATTTAAAGAGC	54	62	XhoI	5261, 307
	**R: TTTGgcGTC** **CTCgAG** **AAT** TTCTTTAAAGATGTCTTTAGAGTGG	54	66		
HP-NAP_Y101H_	**F: ACAAAcAT** **CTcGAg** **AAAGAA** TTTAAAGAGCTCTCTAACACC	54	58	XhoI	5261, 307
	**R: TTCTTT** **cTCgAG** **ATgTTTGT** AGTCCTCTAGAATTTCTTTAAAGA	54	62		
HP-NAP_E103A_	**F: TCTAGcAAAA** **GAATTc** **AAAGA** GCTCTCTAACACCGCTGAAAA	54	62	EcoRI	5568
	**R: TCTTT** **gAATTC** **TTTTgCTAGA** TATTTGTAGTCCTCTAGAATTTCT	54	62		
HP-NAP_E103D_	**F: ACAAATATCTAGAcAAA** **GAATT** c AAAGAGCTCTCTAACACCGCT	54	62	EcoRI	5568
	**R:** **AATTC** **TTTgTCTAGATATTTGT** AGTCCTCTAGAATTTCTTTAAAGA	54	62		
HP-NAP_E97GY101H_	**F: ACAAAcAT** **CTcGAg** **AAAGAA** TTTAAAGAGCTCTCTAACACCG	54	62	XhoI	5261, 307
	**R: TTCTTT** **cTCgAG** **ATgTTTGT** AGTCCcCTAGAATTTCTTTAAAGAT	54	66		

^a^Primer-primer overlapping sequences are written in bold

^b^Inserted silent restriction sites are underlined

^c^Mutations are written in lowercase letters

^d^Tm_pp_ and Tm_no_ were calculated as: Tm = 2 °C x (number of the A and T bases) + 4 °C x (number of the G and C bases)

^e^Tm_pp_ was calculated from the primer-primer overlap sequence

^f^Tm_no_ was calculated from the primer sequence matched to the template

^g^The Tm_no_ value of 62 °C was used for the PCR reaction to generate the mutations if the Tm_no_ values of forward and reverse primers are different

^h^The digested products are DNA fragments from the plasmid with desired mutations after digestion with the inserted silent restriction enzyme

The PCR reactions were carried out with 10 ng of plasmid pET42a-NAP as the DNA template, 1 μM mutagenesis primer pairs, 200 μM deoxynucleoside triphosphates (dNTPs), and 3 units of high-fidelity PCR enzyme mix (Expand Long Template PCR System, Roche) in a final volume of 25 μl. The PCR cycles were initiated at 95 °C for 10 min to denature the template DNA, followed by 14 amplification cycles. Each amplification cycle consisted of 95 °C for 1 min, Tm_no_ – 5 °C for 1 min and 72 °C for 6 min. The PCR cycles were finished with an additional annealing step at Tm_pp_ – 5 °C for 1 min and an extension step at 72 °C for 30 min. The PCR reactions with a volume of 15 μl were treated with 5 units of DpnI (New England Biolabs, NEB) at 37 °C for 2 h. Then, 2 μl of both DpnI-treated and DpnI-untreated PCR reactions was analyzed by agarose gel electrophoresis. As shown in Fig. 2, three major DNA bands appear in the gel for the no-primer control reaction. The middle brightest band represents the supercoiled template DNA (Fig 2). The upper and the lower bands represent the nicked circular and the circular single-stranded plasmid DNA, respectively (Fig. 2). Similar but different patterns of DNA bands were observed for the PCR reactions compared to that for the no-primer control reaction. In the PCR reaction, the desired supercoiled DNA was detected as a band higher than but close to position of the marker band of 6000 base pairs (bp) (Fig. 2). Additional amplification products with sizes higher than the desired supercoiled DNA were also observed (Fig. 2). In the PCR reactions, no circular single-stranded plasmid DNA was detected while primer-dimer was detected as the band size below 250 bp (Fig. 2). After treatment with DpnI, only one major band with the size comparable to that of the supercoiled template DNA was detected for the PCR reactions, whereas no such band was detected for the no-primer control reaction (Fig. 2). Thus, the template DNA plasmid was almost fully digested by DpnI. The PCR products were successfully generated by this modified SDM method.

**Fig. 2:**
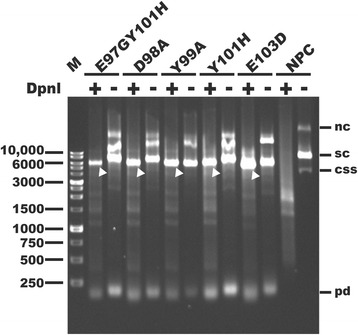
Examination of the efficiency of DpnI digestion of the PCR products. The PCR products of plasmid pET42a-NAP encoding the *napA* gene with the indicated mutations, E97GY101H, D98A, Y99A, Y101H, and E103D, and no-primer PCR control (NPC) were left untreated (−) or treated (+) with restriction enzyme DpnI and then analyzed by 1% agarose gel electrophoresis. Arrows indicate the PCR products resistant to the DpnI digestion. Migration of supercoiled (sc), nicked circular (nc), circular single-stranded (css), and primer-dimer (pd) DNA is indicated. Lane M: 1 kb DNA ladder (SMOBiO, Hsinchu, Taiwan)

Since the designed primers were introduced with the silent restriction sites, the plasmids containing the desired mutation generated from this modified PCR mutagenesis methods can be first screened by silent restriction enzyme digestion. The above DpnI-treated PCR products with a volume of 2 μl were then transformed into *E. coli* DH5α competent cells by heat shock. The plasmid DNA isolated from at least one to four colonies generated by the transformation of each DpnI-treated PCR products was analyzed for the presence of the introduced silent restriction site by a restriction digest. The plasmids of four HP-NAP mutants, HP-NAP_D98A_, HP-NAP_Y99A_, HP-NAP_Y101H_, and HP-NAP_E97GY101H_, were designed to be cleaved twice by the restriction enzyme XhoI to form two bands with sizes of 5261 and 307 bp. As shown in Fig. 3a, the plasmids from the only clone of HP-NAP_D98A_ mutant, all three clones of HP-NAP_Y99A_ mutant, two of the four clones of HP-NAP_Y101H_ mutant, and three of the four clones of HP-NAP_E97GY101H,_ were cleaved by XhoI to form two bands with sizes close to 5200 bp and 300 bp. The plasmids of the other two HP-NAP mutants, HP-NAP_E103A_ and HP-NAP_E103D_, were designed to be cleaved once by the restriction enzyme EcoRI to form a single band of 5568 bp. After treatment with EcoRI, the plasmid from one of the three clones of HP-NAP_E103A_ mutant and one of the two clones of HP-NAP_E103D_ mutant formed a linearized band which can be distinguished from the uncut unmutated parental plasmids (Fig. 3b). Thus, not all the plasmids were successfully inserted with the introduced silent restriction site. For the six HP-NAP mutants, the silent restriction site mutation is present in one-third to all of the clones being screened (Table 2), indicating that the non-mutated template DNA was still present in the PCR products after DpnI digestion. The non-mutated template DNA may be recovered from the incomplete digestion of hemi-methylated DNA molecules, which is more resistant to DpnI digestion [11]. For all six HP-NAP mutants, the plasmid being identified to contain the silent restriction site mutation was found to contain the desired mutation and to have the correct sequence (Table 2). Thus, introduction of a restriction site by silent mutation in the mutated construct does increase the efficiency for mutant screening.

**Fig. 3:**
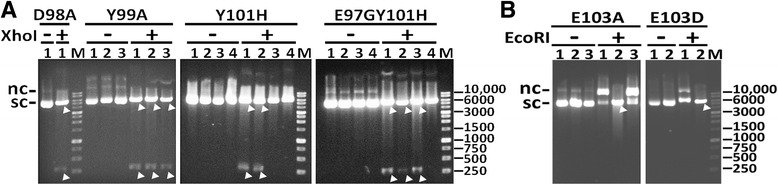
DNA agarose gel analysis of restriction digest of the plasmids ioslated from HP-NAP mutants. The plasmids isolated from the colonies of HP-NAP_D98A_, HP-NAP_Y99A_, HP-NAP_Y101H_, and HP-NAP_E97GY101H_ mutants (**a**) or the colonies of HP-NAP_E103A_ and HP-NAP_E103D_ mutants (**b**) were left untreated (−) or treated (+) with restriction enzymes XhoI (**a**) or EcoRI (**b**), respectively, and then analyzed by 1% agarose gel electrophoresis. The two cut DNA fragments shown in **a** and the linear product shown in **b** are indicated by the white triangles. Migration of supercoiled (sc) and nicked circular (nc) DNA is indicated. Lane M: 1 kb DNA ladder (SMOBiO, Hsinchu, Taiwan)

**Table 2:** Analysis of the Mutagenesis Efficiency of the Modified PCR-based Site-directed Mutagenesis Method

Mutated plasmids	Transformed colonies	Silent restriction enzyme digestion			DNA sequencing		
		Plasmids screened^a^	Desired plasmids	Mutagenesis efficiency^b^	Plasmids screened^a^	Desired plasmids	Mutagenesis efficiency^b^
HP-NAP_D98A_	1	1	1	1/1 (100%)	1	1	1/1 (100%)
HP-NAP_Y99A_	3	3	3	3/3 (100%)	1	1	1/1 (100%)
HP-NAP_Y101H_	4	4	2	2/4 (50%)	1	1	1/1 (100%)
HP-NAP_E103A_	40	3	1	1/3 (33%)	1	1	1/1 (100%)
HP-NAP_E103D_	2	2	2	1/2 (50%)	1	1	1/1 (100%)
HP-NAP_E97GY101H_	28	4	3	3/4 (75%)	1	1	1/1 (100%)

^a^The plasmids screened by silent restriction enzyme digestion were isolated from randomly selected clones, whereas the plasmids screened by DNA sequencing were randomly selected from the desired plasmids identified by silent restriction enzyme digestion

^b^Mutagenesis efficiency was calculated as the number of desired plasmids out of a number of plasmids selected for screening

In this study, we have successfully generated single and double point mutations together with the introduced silent restriction site mutation on HP-NAP by this modified PCR-based SDM method. Usually, mutants are selected by DNA sequencing to check if they contain the desired mutations. If there is a false positive clone resulted from the inefficiently digestion of the template DNA by DpnI after the PCR, one need to send another plasmid for sequence verification. It is time and money consuming. Here, the silent restriction site mutation near to the target mutation site was introduced by using the SDM-assist software to increase the efficiency for mutant screening. Our result shows that the plasmid carrying the introduced silent restriction site mutation does contain the desired mutation. Consistent with our findings, previous report has shown that introduction of a silent restriction site in the primer does provide a convenient and reliable mutant screening in an inverse PCR-based SDM [12]. However, the silent restriction site needs to be introduced in the proximity of the desired mutation site in the primer using their method. In our method, there is no such limitation in the primer design since the silent restriction site mutation can be introduced at either the primer-primer complementary sequences or the non-overlapping sequences. In the other study, a novel critical annealing temperature (Tc)-PCR method has been developed to replace the DpnI digestion for distinguishing desired mutants from parental templates and undesired mutants [13]. However, a gradient PCR with an accurate Tc needs to be performed for the mutant screening. By using our method, the desired mutants can be easily screened and identified by restriction digestion.

The selection of the silent restriction site mutation is critical for analyzing the potential mutant clones. We suggest to introduce the silent restriction mutation that generate a restriction enzyme cleavage site with two cuts on the mutated plasmid to allow the clear identification of the mutant. The size of the cleaved product equal to or larger than 250 bp is preferred since such band will be much easier for visualization on the agarose gel. If the introduced silent restriction site is only present once on the mutated plasmid, it could be difficult to identify the mutated plasmid after restriction enzyme digestion due to the same migration distance of the linearized mutated DNA and the unmutated parental template DNA in the agarose gel. Even if the migration distance is not the same, a complete digestion is required for such distinction to ascertain if the plasmid contains the desired mutation. If the introduced silent restriction site is present many times on the mutated plasmid, the analysis could be complicated due to the presence of too many DNA fragments on the agarose gel.

In conclusion, our approach combining the one-step PCR-based multiple site-directed plasmid mutagenesis method with extended non-overlapping sequence at the 3′ end of the primer and the introduction of silent restriction sites in the primers using the SDM-Assist software provides an additional means to identify the mutation before sequencing validation. This combination approach is suitable for generating single and multiple-site mutations in the genes with low GC content and is more applicable for generating mutated proteins for their functional study.

## Abbreviations

Bp: base pairs
Css: Circular single-stranded
dNTPs: Deoxynucleoside triphosphates
HP-NAP: *Helicobacter pylori* neutrophil-activating protein
Nc: Nicked circular
NPC: No-primer PCR control
PCR: Polymerase chain reaction
Pd: Primer dimer
RE: Restriction enzyme
Sc: Supercoiled
SDM: Site-directed mutagenesis
Tc: Critical annealing temperature
Tm: Melting temperature
